# Characterization of the MeWRKY12–*MeSSIV* Regulatory Module and *MeWRKY12* Promoter Activity in Cassava

**DOI:** 10.3390/genes17101243

**Published:** 2026-10-09

**Authors:** Yu-Qiang Guo, Xiao-Hua Lu, Yi-Heng Wang, Lu-Hua Wang, Yan Wang, Wen-Ya Guo, Rui-Mei Li, Jiao Liu, Yuan Yao, Jian-Chun Guo, Ya-Jie Wang, Meng-Ting Geng

**Affiliations:** 1 National Key Laboratory for Tropical Crop Breeding, School of Life and Health Sciences, Hainan University, Haikou 570228, China; 2 Key Laboratory of Biology and Genetic Resources of Tropical Crops, Ministry of Agriculture, Key Laboratory for Biology and Genetic Resources of Tropical Crops of Hainan Province, Institute of Tropical Bioscience and Biotechnology, Chinese Academy of Tropical Agricultural Sciences, Haikou 571101, China; 19071010110006@hainanu.edu.cn (X.-H.L.);; 3 School of Tropical Agriculture and Forestry, Hainan University, Haikou 570228, China

**Keywords:** cassava, *MeWRKY12*, *MeSSIV*, starch biosynthesis, transcriptional regulation, phytohormone response

## Abstract

Background: Soluble starch synthases are essential for starch formation, but their transcriptional regulation remains poorly understood in cassava. Starch synthase IV (SSIV) is an important enzyme in starch biosynthesis, and this study aimed to investigate the transcriptional regulation of cassava *MeSSIV*. Methods: Yeast one-hybrid, transcriptional activation, subcellular localization, and dual luciferase assays were performed to evaluate MeWRKY12 binding to and activation of the *MeSSIV* promoter. Quantitative real-time PCR was used to analyze *MeWRKY12* expression in different tissues. Histochemical staining and quantitative GUS assays were conducted using *proMeWRKY12* reporter plants grown under field conditions for six months. Promoter responses to abscisic acid (ABA), methyl jasmonate (MeJA), and gibberellic acid (GA_3_) were examined in storage root slices. Results: MeWRKY12 bound to the tested *MeSSIV* promoter region in yeast, exhibited transcriptional activation activity, localized to the nucleus, and enhanced *MeSSIV* promoter activity in plant cells. *MeWRKY12* transcript abundance was highest in storage roots. GUS activity was also highest in storage roots, followed by fibrous roots. Histochemical staining revealed broad promoter activity in vascular, cambial, and parenchymatous tissues, including the xylem parenchyma of storage roots. Promoter activity increased following ABA, MeJA, and GA_3_ treatments, with distinct temporal response patterns. Conclusions: These findings provide evidence at the promoter level supporting MeWRKY12 as a candidate positive regulator of *MeSSIV* transcription and reveal variation in *proMeWRKY12* activity across tissues and hormone treatments. This study provides a basis for investigating WRKY transcription factors in the regulation of starch biosynthesis in cassava.

## 1. Introduction

Cassava (*Manihot esculenta* Crantz) is an important tropical root crop whose starch-rich storage roots are widely used as food, animal feed, and industrial raw materials. Starch accumulation and structure are determined by the coordinated expression and activity of starch biosynthetic enzymes in storage-root amyloplasts. Although several of these enzymes have been characterized in cassava, the transcriptional mechanisms regulating their expression during storage-root development remain poorly understood.

Soluble starch synthases (SSs) catalyze the elongation of α-1,4-linked glucan chains and have distinct roles in determining amylopectin architecture and starch granule properties. In cassava, *MeSSII* regulates amylopectin structure, starch thermal properties, and the assembly of starch-biosynthetic multienzyme complexes [1], whereas *MeSSIII-1* contributes to glucan-chain elongation and amylopectin chain-length distribution [2]. SSIV is involved in starch granule initiation and glucan-chain elongation, although its function varies among plant species. Loss of *AtSSIV* reduces starch granule number and produces fewer but larger granules in *Arabidopsis* chloroplasts [3], whereas overexpression of *AtSSIV* increases starch accumulation in potato tubers [4]. In wheat, mutation of *TaSSIVb-D* alters total starch and amylopectin contents, whereas rice *SSIV-2* alleles affect starch gelatinization and pasting properties [5,6]. However, the function and upstream transcriptional regulation of *MeSSIV* in cassava storage roots remain unclear.

Starch biosynthesis is regulated not only by the catalytic activities and interactions of biosynthetic enzymes but also by transcriptional networks that coordinate gene expression with plant development and environmental signals. Members of the bHLH, bZIP, NAC, and MYB families can directly regulate starch biosynthetic genes in starch-accumulating organs [7]. In cassava, bZIP2 directly binds to and activates the promoters of *APL1*, *ISA1*, and *GBSS1* in the starchy cultivar SC205, whereas these interactions are absent in the sugary cultivar SM, largely because of amino-acid substitutions in the bZIP2 DNA-binding domain, although structural variations in the target promoters may also contribute [8]. Nevertheless, few transcription factors controlling cassava starch biosynthesis have been functionally characterized, and the upstream regulators of cassava soluble starch synthase genes remain largely unknown.

WRKY proteins are plant-specific transcription factors characterized by a conserved WRKYGQK motif and a C_2_H_2_- or C_2_HC-type zinc-finger structure. Most WRKY proteins recognize W-box elements containing the core sequence TTGACC/T and function as transcriptional activators or repressors. Through transcriptional feedback, protein interactions, and post-translational regulation, WRKY factors integrate developmental, environmental, and phytohormonal signals and regulate plant growth, metabolism, pathogen defense, and adaptation to abiotic stress [9,10,11]. However, compared with their established roles in stress responses, the involvement of WRKY proteins in primary carbon metabolism remains less well understood.

An initial genome-wide analysis identified 85 *MeWRKY* genes and revealed tissue-dependent expression and responsiveness to drought, salinity, cold, H_2_O_2_, and ABA [12]. A subsequent analysis identified 102 typical *MeWRKY* members and functionally characterized group IIa members involved in cassava bacterial blight resistance [13]. Functional studies have mainly focused on stress responses. MeWRKY79 activates the melatonin biosynthetic gene *MeASMT2*, while MeWRKY20, MeWRKY75, MeWRKY27, and MeWRKY33 participate in regulatory modules controlling cassava bacterial blight resistance [13,14,15,16]. Cassava WRKY genes have also been implicated in cassava mosaic disease responses [17]. MeWRKY20 is required for drought resistance in cassava [18], whereas MeWRKY30 enhanced drought tolerance when expressed in transgenic Arabidopsis [19]. More recently, overexpression of *MeWRKY30* was shown to enhance cold tolerance in cassava, and MeSR45-2 was found to recruit NuA4-associated components and transcription factors to activate *MeWRKY30* and other cold-responsive genes [20]. Thus, functionally characterized MeWRKY proteins are predominantly associated with biotic and abiotic stress, whereas their roles in cassava starch biosynthesis remain unknown.

In our previous study, we identified putative ABA-, MeJA-, and gibberellin-responsive cis-elements in the *MeWRKY12* promoter and demonstrated that *proMeWRKY12* responds to exogenous phytohormones in 28-day-old in vitro-grown cassava plantlets [21]. However, the downstream targets of MeWRKY12 remain unidentified, and its expression and promoter activity after storage-root formation have not been characterized. Because promoter activity can vary with tissue type, developmental stage, and growth conditions [22], analysis of *proMeWRKY12* in six-month-old field-grown cassava is necessary to clarify the biological context of MeWRKY12 function.

In this study, we investigated the transcriptional relationship between MeWRKY12 and *MeSSIV*. We examined the binding of MeWRKY12 to the *MeSSIV* promoter, its transcriptional activation capacity and subcellular localization, and its effect on *MeSSIV* promoter activity. We further characterized *MeWRKY12* expression and *proMeWRKY12* activity in different organs and tissues of six-month-old field-grown cassava and evaluated the response of *proMeWRKY12* to ABA, MeJA, and GA_3_ in storage roots. Our results identify MeWRKY12 as a positive transcriptional regulator of *MeSSIV* and provide new insight into the transcriptional and phytohormone-responsive regulation of starch biosynthesis in cassava storage roots.

## 2. Materials and Methods

**Plant materials and sampling**

Cassava cultivar SC8 and the previously generated stable *proMeWRKY12*:GUS-GFP transgenic line #1 [21] were used. Non-transgenic SC8 plants served as controls. Plants were grown under field conditions in Sanya, Hainan, China. Young leaves (YL), mature leaves (ML), petioles (P), stems (S), fibrous roots (FR), and storage roots (SR) were collected from six-month-old plants. Three independent plants were sampled as biological replicates for each tissue analysis.

**Yeast one-hybrid assay**

The *MeSSIV* promoter was cloned into the pAbAi vector and integrated into the genome of the Y1HGold yeast strain. The minimum aureobasidin A (AbA) concentration required to suppress basal promoter activity was determined before the interaction assay. The 618 bp full-length coding sequence of *MeWRKY12* was inserted into pGADT7, and the resulting construct was introduced into the bait strain. Yeast transformed with the empty pGADT7 vector was used as a negative control. Transformants were cultured on SD/−Leu−Ura medium supplemented with 200 ng mL^−1^ AbA at 30 °C for 3–4 d. Yeast growth was used to evaluate the interaction between MeWRKY12 and the *MeSSIV* promoter. The experiment was independently repeated three times.

**Transcriptional activation assay**

The full-length coding sequence of *MeWRKY12* was cloned into pGBKT7 and transformed into AH109 yeast cells. Cells carrying the empty pGBKT7 vector were used as the negative control. Transformants were first selected on SD/−Trp medium and subsequently cultured on SD/−Trp medium containing 20 µg/mL X-α-Gal. Colony growth and blue coloration were examined after incubation at 30 °C for 3–4 d to determine the transcriptional activation activity of MeWRKY12.

**Subcellular localization**

The *MeWRKY12* coding sequence without the stop codon was fused in frame with GFP under the control of the CaMV 35S promoter. The MeWRKY12:GFP construct and empty GFP vector were separately introduced into *Agrobacterium tumefaciens* strain GV3101 and transiently expressed in *Nicotiana benthamiana* leaves. GFP fluorescence was observed 36 h after infiltration using a confocal laser-scanning microscope (Nikon AX, Nikon, Tokyo, Japan). The experiment was performed three times with similar results.

**Quantitative real-time PCR analysis of *MeWRKY12* expression**

Young leaves, mature leaves, stems, fibrous roots, and storage roots were collected from six-month-old SC8 plants. Three independent plants were used as biological replicates. Total RNA was extracted using the Plant Total RNA Isolation Kit Plus (FOREGENE, Chengdu, China), and first-strand cDNA was synthesized using the Evo M-MLV Reverse Transcription Kit (Accurate Biology, Changsha, China) according to the manufacturers’ instructions. Quantitative real-time PCR (qRT-PCR) was performed using ChamQ Universal SYBR qPCR Master Mix (Vazyme, Nanjing, China). *MeWRKY12* transcript levels were normalized to the *MeTubulin* gene and calculated using the 2*^−ΔΔCt^* method, with young leaves as the calibrator. Primer sequences are listed in Table 1.

**Dual-luciferase reporter assay**

A 2000-bp fragment of the *MeSSIV* promoter was inserted upstream of the firefly luciferase gene in pGreenII 0800-LUC to generate the reporter construct (*proMeSSIV*-LUC). The *MeWRKY12* coding sequence was cloned into pGreenII 62-SK to produce the effector vector (62SK-*MeWRKY12*), while the empty pGreenII 62-SK vector served as the vector control (62SK). The constructs were introduced into *A. tumefaciens* GV3101 carrying the pSoup helper plasmid and co-infiltrated into *N. benthamiana* leaves. Luciferase activities were measured 48 h after infiltration using a dual-luciferase reporter assay kit (YEASEN Biotechnology (Shanghai) Co., Ltd., Shanghai, China; Cat. No. 11402ES60). Firefly luciferase activity was normalized to Renilla luciferase activity, and promoter activity was expressed as the LUC/REN ratio. Three independent biological replicates were analyzed.

**Histochemical GUS staining and tissue localization**

Histochemical GUS staining was performed using samples collected from six-month-old field-grown non-transgenic SC8 and plants of the previously reported *proMeWRKY12*:GUS-GFP transgenic line #1 [21]. Young and mature leaves, petioles, stems, fibrous roots, and storage roots were analyzed. Samples from non-transgenic SC8 plants were processed in parallel as negative controls. GUS staining was conducted as previously described [21], with minor modifications. Samples were immersed in GUS staining solution, vacuum-infiltrated, and incubated at 37 °C in darkness for approximately 12 h. Chlorophyll was removed from leaf and stem samples using 70% ethanol. Fresh hand-cut sections of petioles, stems, and storage roots were stained using the same procedure and examined under a stereomicroscope (Nikon SMZ18, Nikon, Tokyo, Japan) to determine the tissue-level distribution of *proMeWRKY12* activity.

**Quantification of GUS activity**

GUS activity was measured in young leaves, mature leaves, petioles, stems, fibrous roots, and storage roots using a commercial GUS activity assay kit (Jiangsu Meimian industrial Co., Ltd., Yancheng, China, MM-6253601) according to the manufacturer’s instructions. Approximately 50 mg of fresh tissue was homogenized in extraction buffer and centrifuged to obtain the soluble protein fraction. Enzyme activity was determined spectrophotometrically and normalized to tissue fresh weight. GUS activity was expressed as U g^−1^ fresh weight. Samples from three independent plants were analyzed.

**Phytohormone treatments**

Storage roots of comparable size were collected from six-month-old field-grown *proMeWRKY12*:GUS-GFP plants. Transverse slices of approximately 3 mm were prepared from the middle region of each storage root and treated separately with 100 μM abscisic acid (ABA), 100 μM methyl jasmonate (MeJA), or 100 μM gibberellic acid (GA_3_). Samples collected at 0 h served as the baseline for comparisons across treatment times. The slices were incubated under controlled conditions at 28 °C and collected at 0, 2, 6, 12, and 24 h after treatment. At each time point, one portion of the material was subjected to histochemical GUS staining, while the remaining tissue was immediately frozen in liquid nitrogen and used for quantitative determination of GUS activity. Three independent storage roots were used for each treatment.

## 3. Results

### 3.1. MeWRKY12 Binds to the MeSSIV Promoter and Functions as a Nuclear Transcriptional Activator

**Figure 1:**
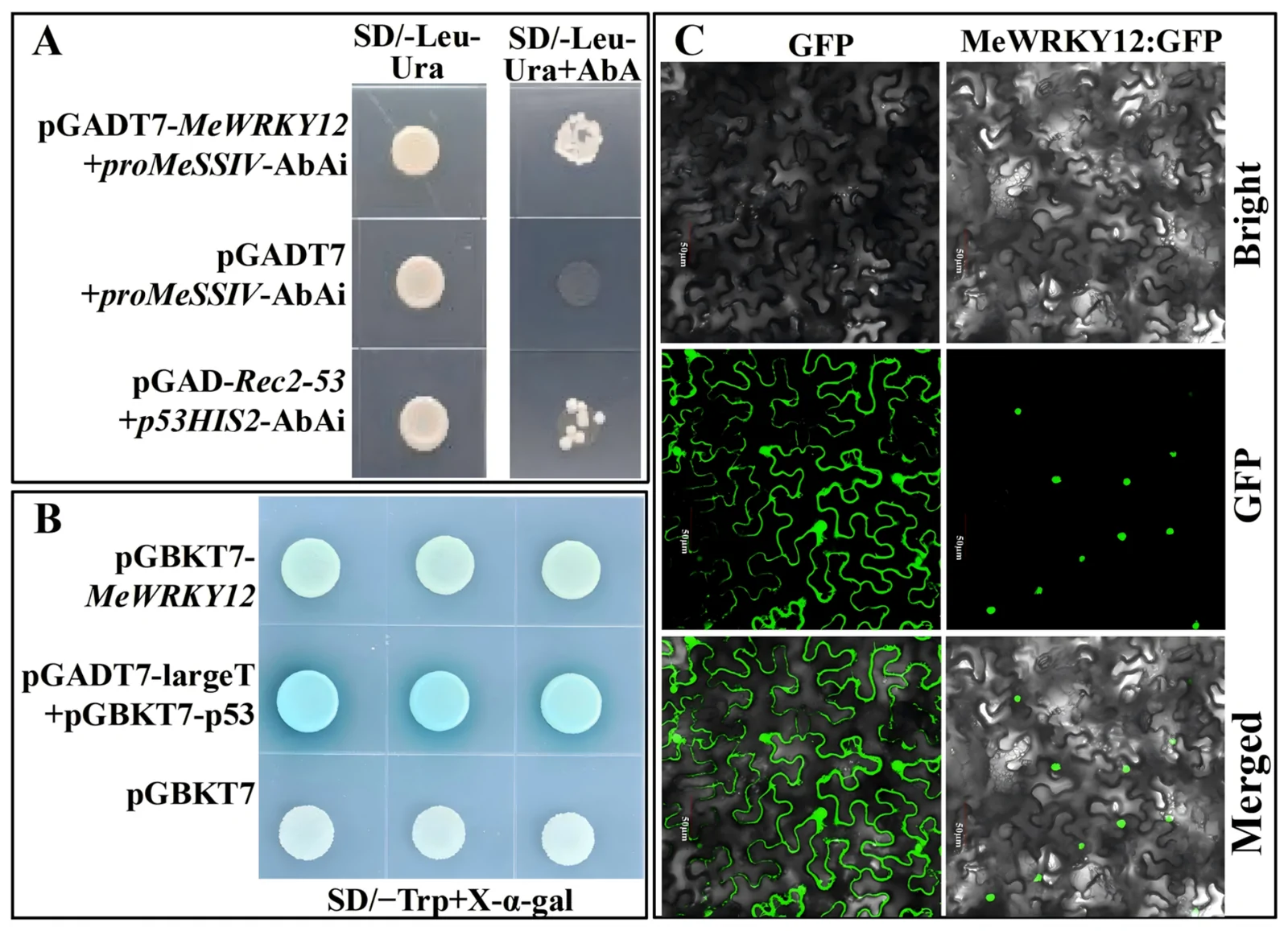
MeWRKY12 binds to the *MeSSIV* promoter and functions as a nuclear transcriptional activator. (**A**) Yeast one-hybrid assay showing the interaction between MeWRKY12 and the *MeSSIV* promoter. The corresponding empty-vector combination was used as the negative control. The concentration of AbA is 200 ng/mL. (**B**) Transcriptional activation activity of full-length MeWRKY12 in yeast. The concentration of X-α-gal is 20 μg/mL. (**C**) Subcellular localization of MeWRKY12:GFP in plant epidermal cells. Bright-field images, GFP fluorescence, and merged images are shown. The empty GFP vector was used as the control. Scale bars = 50 μm.

To identify transcription factors regulating *MeSSIV*, a yeast one-hybrid assay was performed using the *MeSSIV* promoter as bait. MeWRKY12 activated the reporter driven by the *MeSSIV* promoter, whereas no reporter activation was detected in the negative control, indicating that MeWRKY12 binds to the *MeSSIV* promoter in yeast (Figure 1A).

The transcriptional activity of MeWRKY12 was subsequently examined in yeast. Full-length MeWRKY12 activated reporter expression compared with the empty-vector control, demonstrating that MeWRKY12 possesses transcriptional activation activity (Figure 1B). Subcellular localization analysis further showed that the MeWRKY12:GFP signal was predominantly detected in the nucleus, whereas the GFP control displayed a broader cellular distribution (Figure 1C). Together, these results indicate that MeWRKY12 is a nuclear transcriptional activator capable of binding to the *MeSSIV* promoter.

### 3.2. MeWRKY12 Is Highly Expressed in Storage Roots and Activates the MeSSIV Promoter

**Figure 2:**
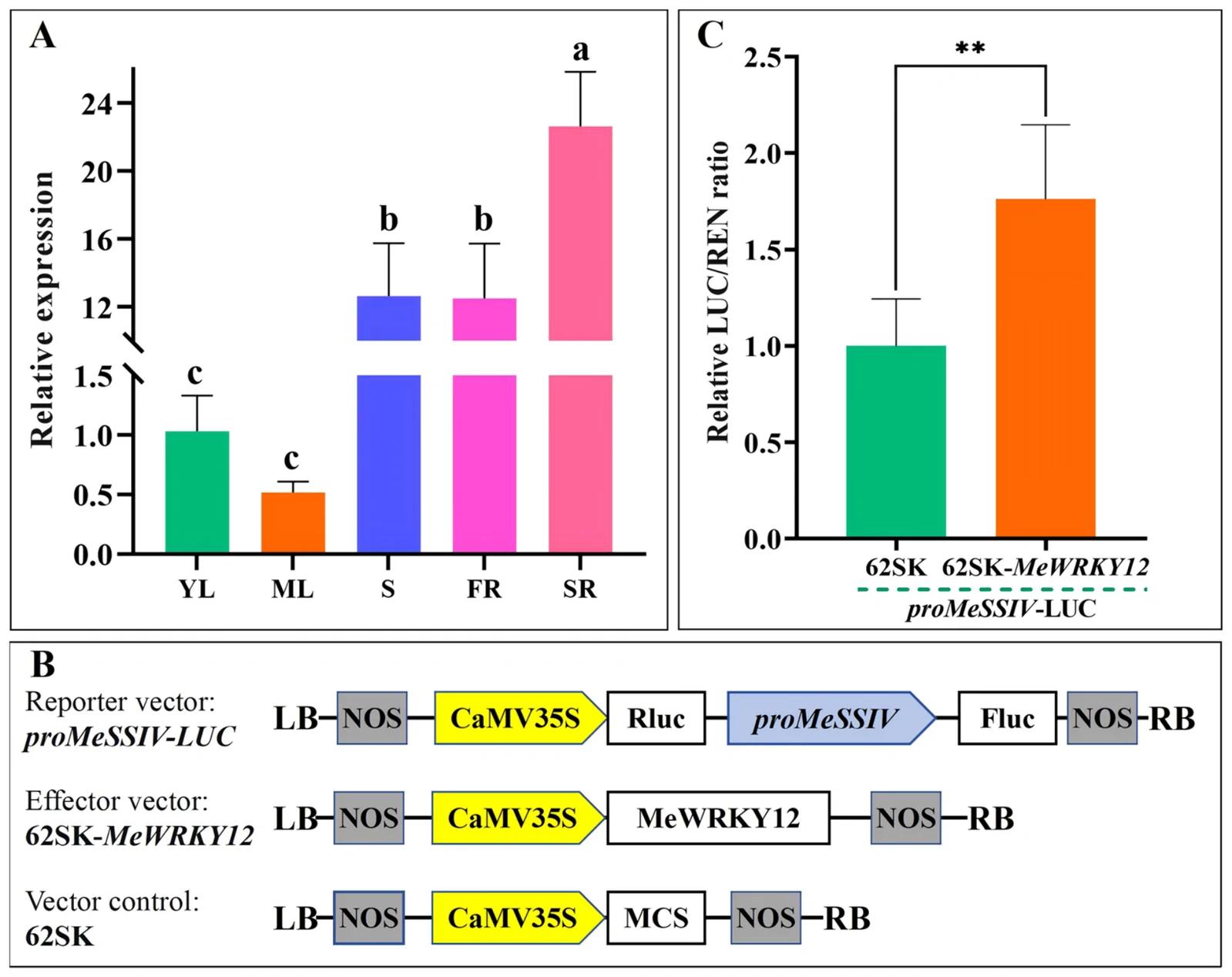
Tissue expression pattern of *MeWRKY12* and activation of the *MeSSIV* promoter by MeWRKY12. (**A**) Transcript abundance of *MeWRKY12* in different cassava tissues. YL, young leaf; ML, mature leaf; S, stem; FR, fibrous root; SR, storage root. Values are means ± SD of three biological replicates. Different lowercase letters indicate significant differences among tissues (one-way ANOVA followed by Tukey’s multiple-range test, *p* < 0.05). (**B**) Schematic representation of the dual-luciferase reporter construct. Rluc, Renilla luciferase; Fluc, firefly luciferase; MCS, multiple cloning site; LB and RB, left and right T-DNA borders; NOS, nopaline synthase terminator. (**C**) Dual-luciferase assay showing activation of the *MeSSIV* promoter by MeWRKY12. The *proMeSSIV*-LUC reporter was co-expressed with either the 62SK empty vector or 62SK-MeWRKY12. Promoter activity is expressed as the relative LUC/REN ratio. Values are means ± SD of three biological replicates. Asterisks indicate a significant difference from the empty-vector control (** *p* < 0.01, two-tailed Student’s *t*-test).

The tissue expression pattern of *MeWRKY12* was examined by qRT-PCR in young leaves, mature leaves, stems, fibrous roots, and storage roots (Figure 2A). *MeWRKY12* expression was highest in storage roots, followed by stems and fibrous roots, with no significant difference between the latter two tissues. Expression was significantly lower in young and mature leaves, which did not differ significantly from each other.

A dual-luciferase reporter assay was performed to determine whether MeWRKY12 regulates *MeSSIV* promoter activity. Co-expression of 62SK-MeWRKY12 with the *proMeSSIV*-LUC reporter increased the relative LUC/REN ratio to approximately 1.76-fold that of the 62SK empty-vector control (Figure 2B,C). Thus, MeWRKY12 significantly activates the *MeSSIV* promoter. Together with the yeast one-hybrid results, these findings identify MeWRKY12 as a positive transcriptional regulator of *MeSSIV*.

### 3.3. proMeWRKY12 Shows Preferential Activity in Roots of Six-Month-Old Field-Grown Cassava

**Figure 3:**
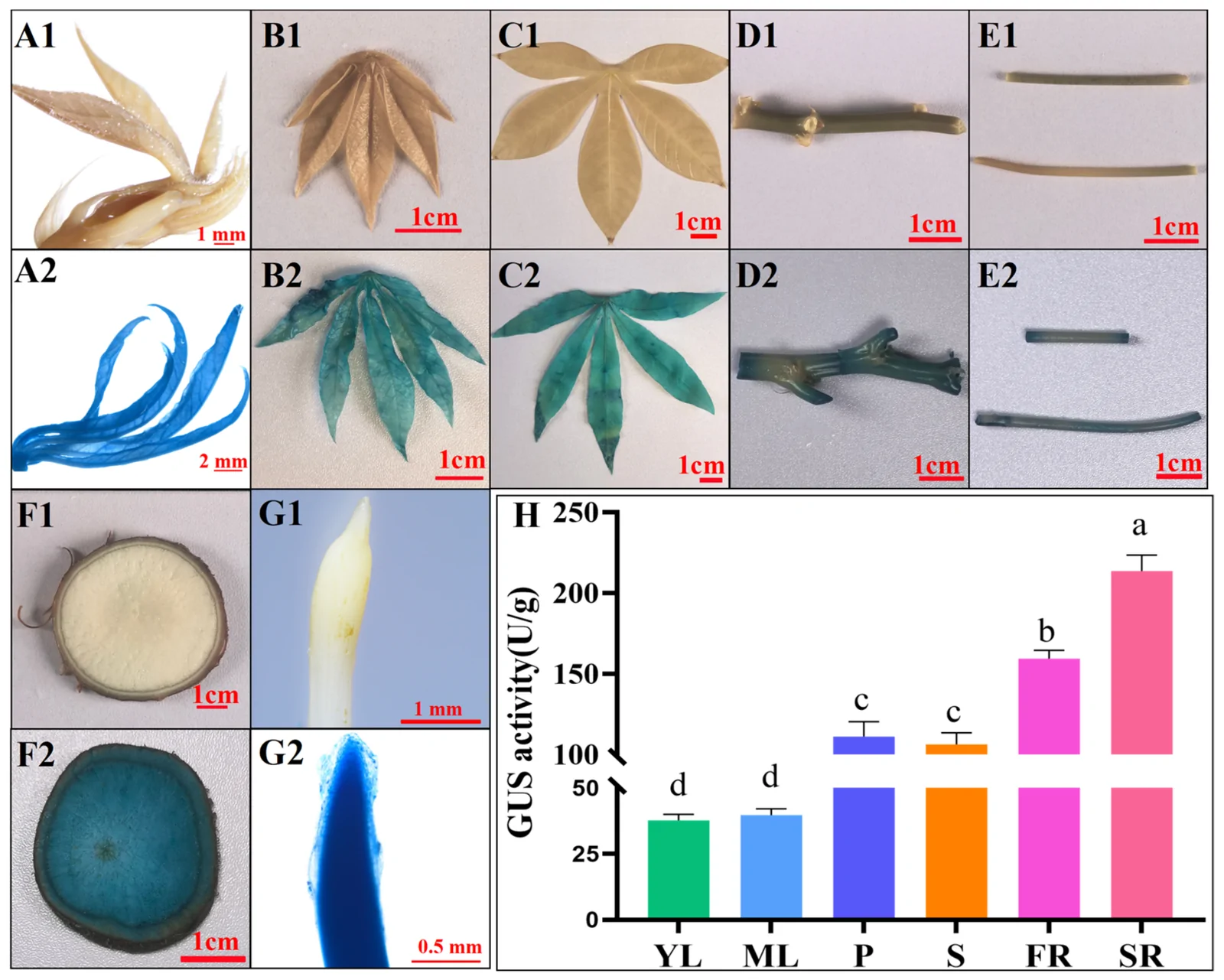
Organ-level activity of *proMeWRKY12* in six-month-old field-grown cassava. (**A**–**G**) Representative GUS staining patterns in emerging leaves (**A**), young leaves (**B**), mature leaves (**C**), stems (**D**), petioles (**E**), storage-root transverse sections (**F**), fibrous roots (**G**). Panels designated “1” show the corresponding organs of non-transgenic SC8 plants, and panels designated “2” show organs from *proMeWRKY12*:*GUS* plants. Scale bars are indicated in the panels. (**H**) Quantification of GUS activity in different organs. YL, young leaf; ML, mature leaf; P, petiole; S, stem; FR, fibrous root; SR, storage root. Values are means ± SD of three biological replicates. Different lowercase letters indicate significant differences among organs (one-way ANOVA followed by Tukey’s multiple-range test, *p* < 0.05).

The cloning and initial characterization of *proMeWRKY12* in 28-day-old in vitro-grown cassava plantlets were reported previously [21]. To characterize its spatial activity under field conditions, stable *proMeWRKY12*:*GUS* reporter plants were grown in the field for six months. Emerging leaves, young leaves, mature leaves, stems, petioles, fibrous roots, and storage roots were collected for histochemical and quantitative analyses (Figure 3).

No detectable blue staining was observed in the corresponding organs of non-transgenic SC8 plants. By contrast, GUS staining was detected in all examined organs of the *proMeWRKY12*:*GUS* plants, although the staining intensity varied markedly among organs (Figure 3A–G). Emerging leaves, young and mature leaves showed relatively weak staining, whereas petioles and stems exhibited readily detectable GUS signals. Strong staining was observed in fibrous roots and storage-root transverse sections.

Quantitative measurement of GUS activity was consistent with the histochemical staining patterns (Figure 3H). Storage roots displayed the highest GUS activity, at 213.7 U g^−1^, followed by fibrous roots at 159.5 U g^−1^. Intermediate activities of 106.2–111.6 U g^−1^ were detected in stems and petioles, whereas young and mature leaves exhibited the lowest activities, at 37.7–39.7 U g^−1^. GUS activity was significantly higher in storage roots than in all other organs, and that in fibrous roots was significantly higher than in the aerial organs.

These results indicate that *proMeWRKY12* is not organ-specific but exhibits preferential activity in roots, particularly storage roots, under field conditions. This activity pattern is broadly consistent with the preferential accumulation of endogenous *MeWRKY12* transcripts in storage roots.

### 3.4. Histochemical Analysis Reveals Broad proMeWRKY12 Activity in Vascular and Storage-Root Tissues

**Figure 4:**
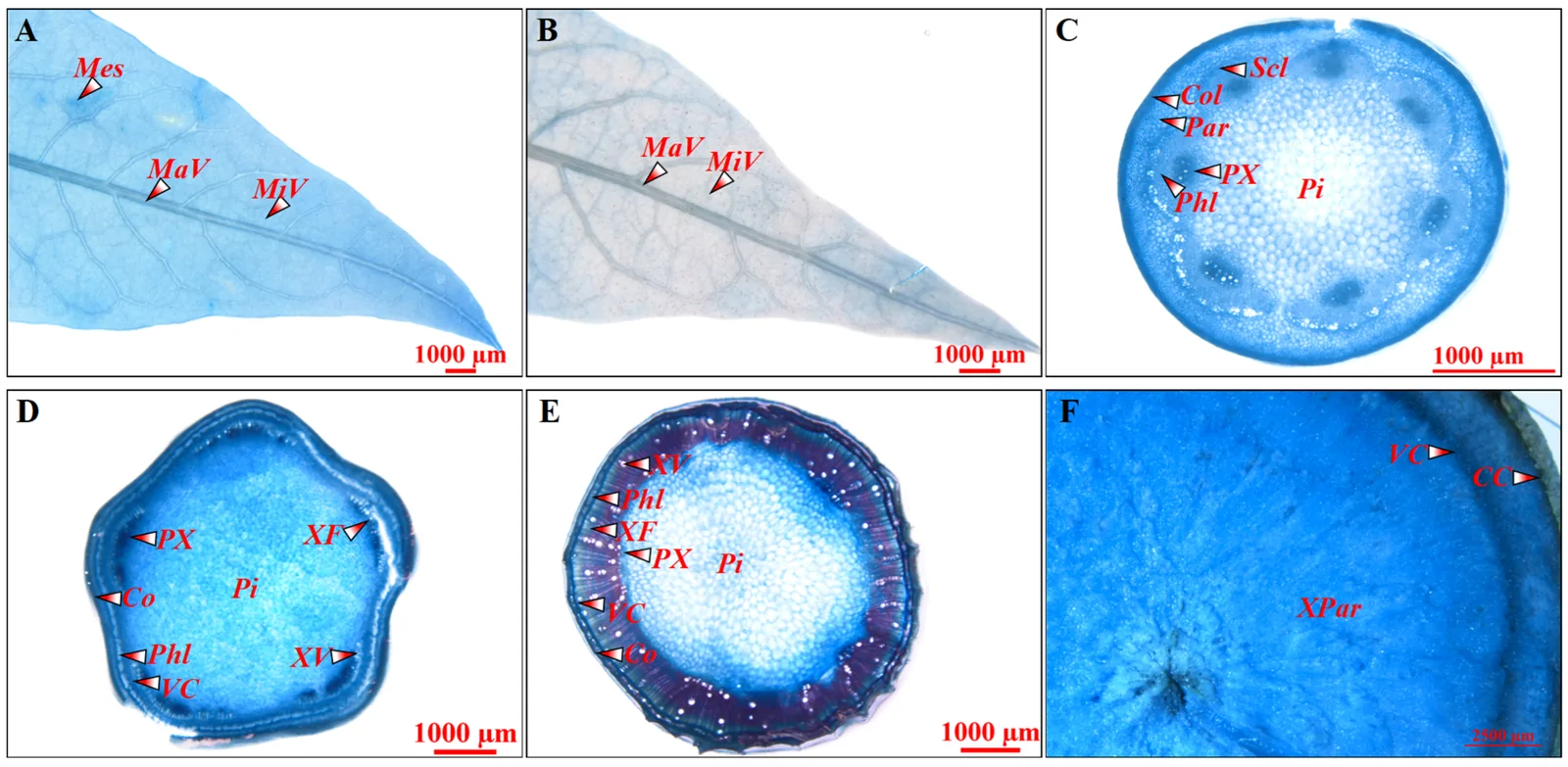
Histochemical localization of *proMeWRKY12* activity in field-grown cassava. Representative GUS staining patterns in tissue sections collected from six-month-old field-grown *proMeWRKY12*:*GUS* plants. (**A**) Mature leaf. (**B**) Young leaf. (**C**) Petiole transverse section. (**D**) Upper-stem transverse section. (**E**) Lower-stem transverse section. (**F**) Storage-root section. CC, cork cambium; Co, cortex; Col, collenchyma; MaV, major vein; Mes, mesophyll; MiV, minor vein; Par, parenchyma; Phl, phloem; Pi, pith; PX, protoxylem; Scl, sclerenchyma; VC, vascular cambium; XF, xylem fiber; XV, xylem vessel; XPar, xylem parenchyma. Scale bars = 1000 μm in (**A**–**E**) and 2500 μm in (**F**).

To characterize the tissue-level activity of *proMeWRKY12*, leaves and sections of petioles, upper stems, lower stems, and storage roots collected from six-month-old field-grown reporter plants were examined by histochemical GUS staining (Figure 4). In mature leaves, GUS staining was observed in the mesophyll and major and minor veins. Young leaves exhibited relatively weak staining, which was mainly distributed along the major and minor veins and their surrounding tissues (Figure 4A,B). In petiole transverse sections, GUS staining was broadly distributed across the collenchyma, sclerenchyma, parenchyma, phloem, and protoxylem (Figure 4C). In upper stems, GUS staining was detected in the cortex, phloem, vascular cambium, protoxylem, xylem fibers, xylem vessels, and pith (Figure 4D). Lower stems showed pronounced staining in the vascular cambium and adjacent phloem and xylem regions, with additional staining in the pith (Figure 4E). In storage-root sections, GUS staining was detected in the cork cambium, vascular cambium, and parenchymatous tissues (Figure 4F). Collectively, these results demonstrate that *proMeWRKY12* exhibits broad but non-uniform activity across cassava tissues, with evident activity in vascular, cambial, and parenchymatous tissues.

### 3.5. ABA Induces proMeWRKY12 Activity in Storage Roots

Previous analysis of 28-day-old in vitro-grown plantlets showed that *proMeWRKY12* responds to phytohormone treatments [21]. To investigate its ABA responsiveness in storage roots, transverse storage-root slices were collected from six-month-old field-grown *proMeWRKY12*:GUS plants and treated with ABA for 0, 2, 6, 12, and 24 h.

**Figure 5:**
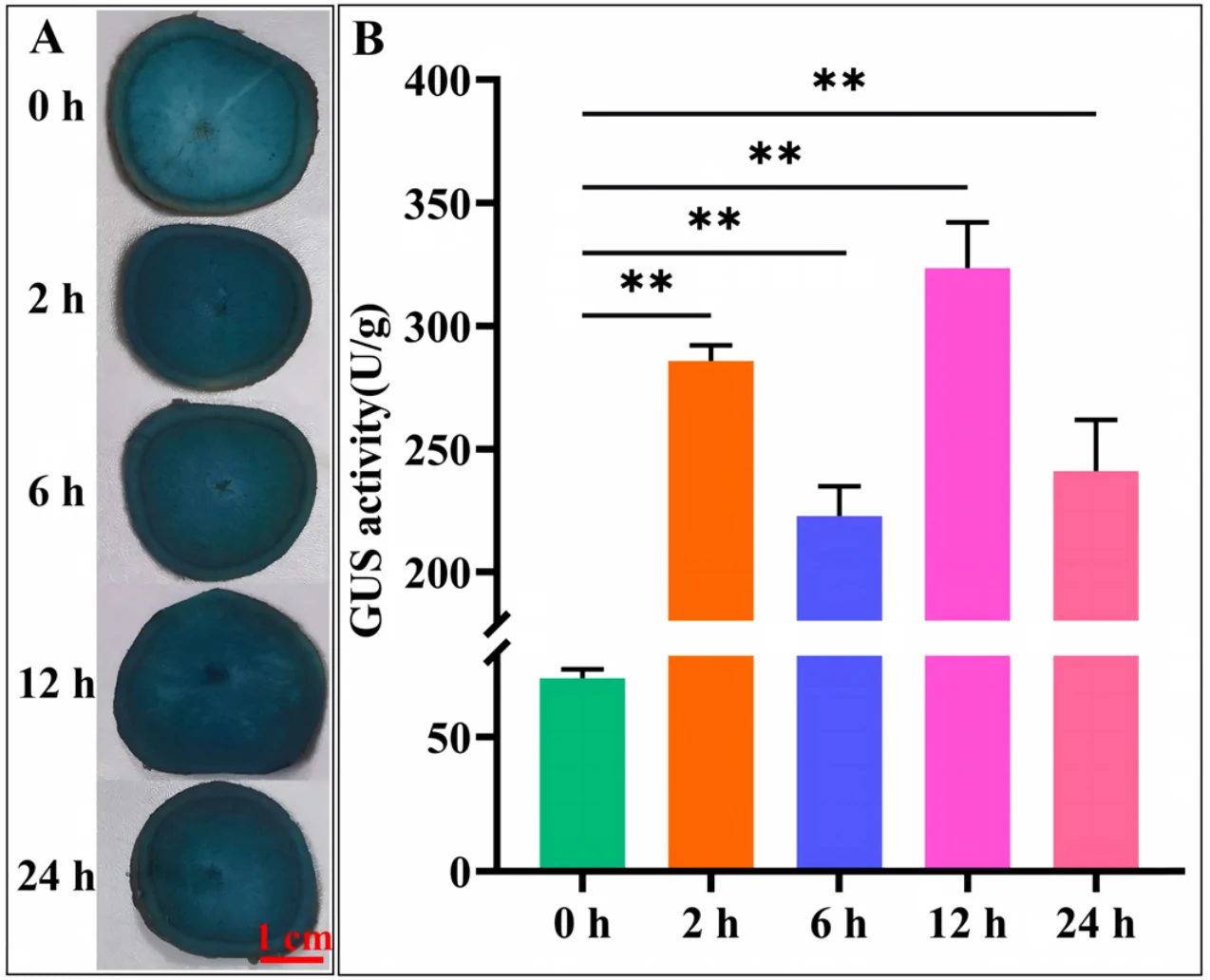
ABA induction of *proMeWRKY12* activity in cassava storage roots. Transverse storage-root slices collected from six-month-old field-grown *proMeWRKY12*:GUS plants were treated with ABA for 0, 2, 6, 12, and 24 h. (**A**) Representative histochemical GUS staining. Scale bar = 1 cm. (**B**) Quantification of GUS enzymatic activity. Values are means ± SD of three biological replicates. Asterisks indicate values significantly different from that at 0 h according to two-tailed Student’s *t*-test (** *p* < 0.01).

Histochemical staining revealed an overall increase in GUS signals following ABA treatment, although the intensity varied over time (Figure 5A). GUS activity increased rapidly following ABA treatment, reaching 285.8 U g^−1^ at 2 h (Figure 5B). It subsequently decreased to approximately 222.6 U g^−1^ at 6 h before reaching approximately 323.4 U g^−1^ at 12 h, the highest level observed during the treatment. At 24 h, GUS activity decreased to approximately 240.9 U g^−1^ but remained significantly higher than that at 0 h.

These results demonstrate that ABA induces *proMeWRKY12* activity in storage roots. GUS activity varied over time following ABA treatment, with relatively high values observed at 2 and 12 h.

### 3.6. MeJA Induces a Dynamic proMeWRKY12 Response in Storage Roots

**Figure 6:**
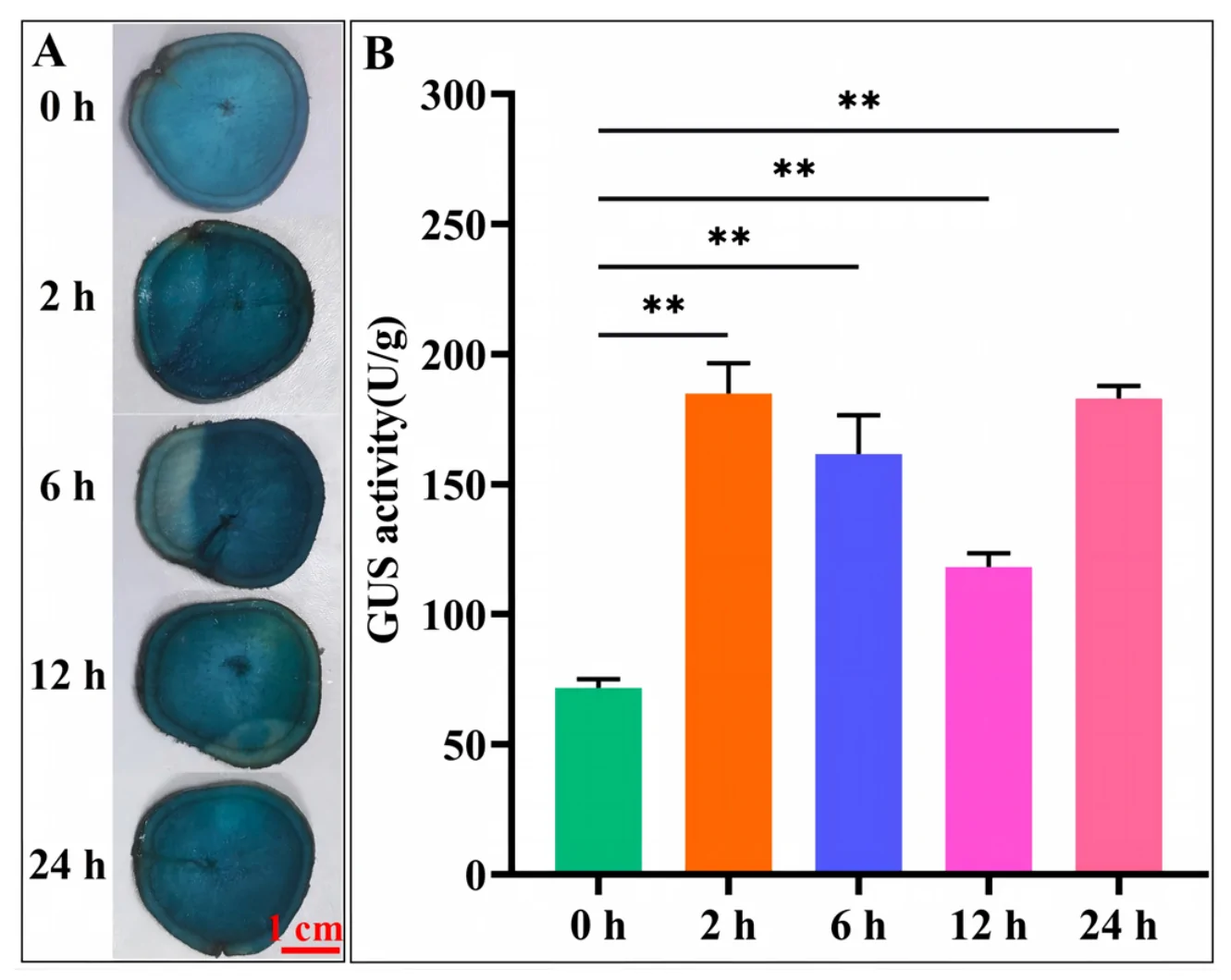
MeJA induction of *proMeWRKY12* activity in cassava storage roots. Transverse storage-root slices collected from six-month-old field-grown *proMeWRKY12:GUS* plants were treated with methyl jasmonate (MeJA) for 0, 2, 6, 12, and 24 h. (**A**) Representative histochemical GUS staining. Scale bar = 1 cm. (**B**) Quantification of GUS enzymatic activity. Values are means ± SD of three biological replicates. Asterisks indicate values significantly different from that at 0 h according to two-tailed Student’s *t*-test (** *p* < 0.01).

MeJA treatment also markedly altered *proMeWRKY12* activity in storage-root slices (Figure 6). Following MeJA treatment, GUS activity increased to 184.9 U g^−1^ at 2 h. Activity remained relatively high at 6 h, at approximately 161.6 U g^−1^, and subsequently decreased to approximately 118.2 U g^−1^ at 12 h. At 24 h, GUS activity increased again to approximately 182.9 U g^−1^ (Figure 6B).

These findings demonstrate that MeJA induces *proMeWRKY12* activity in storage roots, with a rapid early response followed by a transient decrease and subsequent recovery. This temporal pattern differs from that previously observed after whole-plant treatment of in vitro-grown plantlets, suggesting that the MeJA responsiveness of *proMeWRKY12* may depend on tissue context, plant developmental stage, or treatment conditions.

### 3.7. GA_3_ Rapidly Induces proMeWRKY12 Activity in Storage Roots

**Figure 7:**
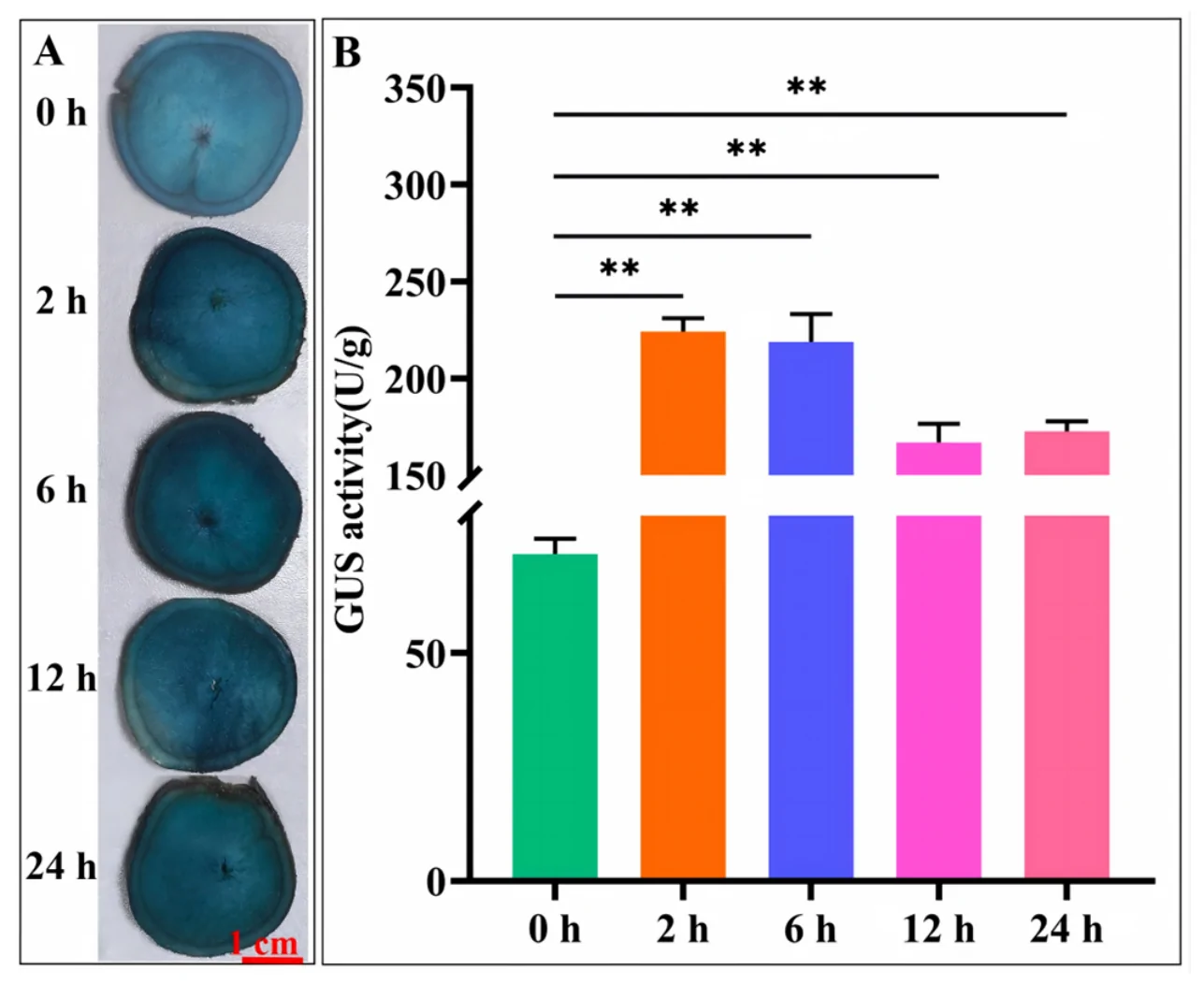
GA_3_ induction of *proMeWRKY12* activity in cassava storage roots. Transverse storage-root slices collected from six-month-old field-grown *proMeWRKY12*:GUS plants were treated with gibberellic acid (GA_3_) for 0, 2, 6, 12, and 24 h. (**A**) Representative histochemical GUS staining. Scale bar = 1 cm. (**B**) Quantification of GUS enzymatic activity. Values are means ± SD of three biological replicates. Asterisks indicate values significantly different from that at 0 h according to two-tailed Student’s *t*-test (** *p* < 0.01).

GA_3_ treatment caused a pronounced increase in GUS staining and enzymatic activity in storage-root slices (Figure 7). Following GA_3_ treatment, GUS activity increased to 224.1 U g^−1^ at 2 h, representing an approximately threefold induction (Figure 7B). A similarly high activity of approximately 218.7 U g^−1^ was maintained at 6 h. Activity subsequently decreased to approximately 166.9 and 172.6 U g^−1^ at 12 and 24 h, respectively. Nevertheless, both values remained significantly higher than that at 0 h.

These results indicate that GA_3_ rapidly induces *proMeWRKY12* activity in storage roots, with the strongest response occurring within 2–6 h. Together, the ABA, MeJA, and GA_3_ treatments demonstrate that *proMeWRKY12* responds to multiple phytohormones in cassava storage roots, although the temporal response differs among hormones.

## 4. Discussion

This study identifies MeWRKY12 as an upstream transcriptional activator of the cassava soluble starch synthase gene *MeSSIV*. MeWRKY12 bound to the tested *MeSSIV* promoter region in yeast, exhibited transcriptional activation activity, localized to the nucleus, and enhanced *MeSSIV* promoter activity in plant cells. Moreover, qRT-PCR showed the highest *MeWRKY12* expression in storage roots, while *proMeWRKY12* reporter activity was strong in fibrous roots and storage roots of six-month-old field-grown cassava plants. These results establish a transcriptional link between MeWRKY12 and *MeSSIV* and suggest that this regulatory relationship may operate during storage-root development.

Although the functions of several cassava soluble starch synthases have been characterized, little is known about their upstream transcriptional regulation. MeSSII and MeSSIII-1 have distinct roles in amylopectin chain elongation, multienzyme-complex formation, and starch structural properties [1,2]. In other species, SSIV participates in starch-granule initiation, glucan-chain elongation, and starch accumulation, although its contribution varies among plant species and storage organs [3,4,5,6]. Transcription factors from several families regulate starch biosynthetic genes, but functionally characterized cassava WRKY proteins have mainly been associated with pathogen defense and abiotic-stress responses [13,14,15,16,17,18,19,20]. The identification of the MeWRKY12–*MeSSIV* relationship therefore extends the known functions of cassava WRKY proteins to the transcriptional regulation of a starch biosynthetic gene.

The yeast one-hybrid and dual-luciferase assays provide complementary evidence for this regulatory relationship. The former showed that MeWRKY12 recognizes the tested *MeSSIV* promoter region, whereas the latter demonstrated its ability to activate this promoter in plant cells. Together with its nuclear localization and transcriptional activation activity, these findings support the role of MeWRKY12 as a positive regulator of *MeSSIV* transcription. WRKY proteins generally recognize W-box elements containing the TTGACC/T core sequence, although binding specificity is also influenced by the surrounding sequence and promoter context [9,10]. Identification of the precise MeWRKY12-binding site through promoter mutagenesis, electrophoretic mobility shift assays, and chromatin immunoprecipitation will be required to define the molecular basis of this interaction in cassava.

The expression pattern of *MeWRKY12* and the spatial activity of *proMeWRKY12* are consistent with a potential role in storage-root development and starch formation. *MeWRKY12* expression measured by qRT-PCR was highest in storage roots, and quantitative GUS analysis likewise showed the highest *proMeWRKY12* reporter activity in storage roots, followed by fibrous roots. At the tissue level, GUS staining was broadly detected in the vascular and cambial tissues of petioles and stems. In storage roots, promoter activity was detected in the cork cambium, vascular cambium, and xylem parenchyma (XPar). The vascular and cambial distribution is consistent with processes related to assimilate transport and secondary growth, whereas xylem parenchyma constitutes the major starch-storing tissue in cassava storage roots [22]. Therefore, the spatial activity of *proMeWRKY12*, particularly its activity in storage-root xylem parenchyma, is compatible with a potential role of MeWRKY12 in storage-root development and starch formation. Previous analysis of 28-day-old in vitro-grown plantlets showed relatively high *proMeWRKY12* activity in young leaves and roots [21], whereas the present study detected its highest activity in storage roots of six-month-old field-grown plants. This difference may reflect variation in plant age, tissue identity, storage-root formation, and growth environment, suggesting that *proMeWRKY12* activity is dependent on developmental and experimental context.

The responses of *proMeWRKY12* to ABA, MeJA, and GA_3_ indicate that its activity is also modulated by phytohormones in storage-root tissues. ABA rapidly increased promoter activity, with the strongest response observed at 12 h, whereas GA_3_ induced a rapid response that peaked at 2–6 h and subsequently declined. MeJA also enhanced promoter activity, although the magnitude of the response varied over time. These temporal patterns are consistent with the predicted ABA-, MeJA-, and gibberellin-responsive elements in the *MeWRKY12* promoter [21]. Hormonal regulation of starch metabolism has also been reported in cassava. The ABA-responsive MeSnRK2.3-MebHLH68 module regulates starch biosynthetic genes, and *MeSnRK2.3* overexpression increases storage-root starch content [23]. ABA and MeJA also induce expression of the cassava starch synthase gene *MeSSIII-1* [24]. In postharvest cassava root slices, MeJA treatment increased starch content relative to controls at 12 and 24 h, but this effect was not sustained at 48 h [25]. In sweet potato, however, GA_3_ application reduced root starch accumulation and downregulated starch biosynthetic genes [26]. These findings highlight the context-dependent effects of phytohormones on starch metabolism. Consistent with this context dependence, the MeJA response observed in storage-root slices differed from the decline in GUS staining previously observed in whole in vitro-grown plantlets. This difference may result from variation in developmental stage, tissue type, treatment method, or the quantitative versus qualitative nature of the assays, suggesting that the hormone responsiveness of *proMeWRKY12* is tissue- and developmental-context dependent. However, whether these hormones influence *MeSSIV* expression through MeWRKY12 remains to be established.

The present results demonstrate the binding and activation of the *MeSSIV* promoter by MeWRKY12 but do not yet establish whether MeWRKY12 affects starch biosynthesis in planta. Genetic analyses using *MeWRKY12* overexpression and knockout lines will be required to determine its effects on *MeSSIV* expression, starch accumulation, amylopectin structure, and starch-granule development. Promoter-element mutagenesis and chromatin-binding analyses will further validate this regulatory relationship under native conditions. In summary, MeWRKY12 is a nuclear transcriptional activator that binds to and activates the *MeSSIV* promoter. The preferential expression of *MeWRKY12* in storage roots and the relatively strong activity of *proMeWRKY12* in fibrous roots and storage roots are consistent with its potential role in regulating *MeSSIV* transcription. The dynamic responses of *proMeWRKY12* to ABA, MeJA, and GA_3_ further suggest that *MeWRKY12* expression may be modulated by phytohormone signaling. These findings broaden the known functions of cassava WRKY transcription factors and provide a basis for elucidating the transcriptional regulatory network of starch biosynthesis in cassava.

## Figures and Tables

**Table 1:** Primers for qRT-PCR amplification.

Gene	Forward Primer (5′ to 3′)	Reverse Primer (5′ to 3′)
*MeWRKY12*	TCCTTCACACTCACACCACTC	TGATCGTTATGAGTACTGAATCCCA
*MeTubulin*	ATGCGGTTCTTGATGTTGTTC	TCGGTGAAGGGAATACAGAGA

## Data Availability

The original contributions presented in this study are included in the article. Further inquiries can be directed to the corresponding authors.
